# Supplementary material for: Lympho-Hematopoietic Microenvironments and Fish Immune System
Source: Biology (Basel). 2022 May 13;11(5):747. doi: 10.3390/biology11050747 (PMC9138301; doi:10.3390/biology11050747)
Supplement: Supplementary file 1 [file biology-11-00747-s001.zip › biology-1697763-supplementary.pdf]

Supplementary Materials

Table S1: Immunocompetent cells in teleost fish.

HSC-independent T lymphoid progenitors		Zebrafish larvae	12
T lymphocytes express:			
TCR-CD3 complex, Rag		Zebrafish, turbot	45, 47, 64, 65
Co-receptors CD4, CD8		Diverse teleosts	64, 65
Co-stimulatory molecules (CD28, CTLA-4, CD80/CD86)		Diverse teleosts	64, 65
CD4 lymphocytes			
CD4.1$^+$ cell include TH1-like cells, TH17 like cells and			64, 67
	Treg (Foxp3$^+$, TGFβ, IL10) cells	Diverse teleosts	67, 127
(abundant in lamina propria zebrafish (97, 163) but low numbers in sea bass (164))			
CD4.2$^+$ cell include TH2-like cells (IL15Rα$^+$ cells) that	predominate in gills, and	Pufferfish	163
	Treg (Foxp3$^+$) cells	Tetraodon	69
Cytotoxic CD8α$^+$ cells	Present in spleen and kidney	Crucian carp, rainbow trout	125, 126
B-lymphocytes			
IgM$^+$, IgD$^+$ cells	Present in kidney (developing and mature) and spleen (mature)	Diverse teleosts	128
IgT$^+$ cells	Mucosae: IgT1-T3	Rainbow trout	24, 179
	IgT/Z1, IgT/Z2	Zebrafish	186
LT-PC	Migrate into pronephros	Trout, cattish, salmonids	129-133
DCs/Langerhans cells	Thymic medulla, kidney, spleen, skin	*Dicentratus*, rainbow trout, salmon	38, 87, 159-162

Table S2: Some molecules involved in T and B cell development in teleosts.

Foxn1, Foxn4	Both transcription factors govern TEC development	Zebrafish	101-103
Dll4	Thymic cortex marker	Medaka	49, 79
Notch1a, 1b, 2, 3	Thymocyte differentiation	Medaka	79
IL2, IL15	Both cytokines signal through IL15R	Tetraodon, trout	69, 81
IL7	Limited immunological role	Zebrafish	82
Aire	Thymic medulla marker	Medaka, zebrafish, pufferfish	49, 90
β5t subunits	Antigen processing in thymus and positive selection	Diverse bony fish	88
Protease Prss16	Antigen processing in thymus and positive selection	Diverse bony fish	88
CCL25 and CXCL12	Thymic seeding	Zebrafish	75, 77
CCR9a	Lymphoid progenitor cell migration into thymus	Medaka	78
CCR9b	Positioning and progression of thymocyte in thymic subcapsulary zone	Medaka	78
CCL20-L1a	B lymphocyte migration from pronephros to posterior kidney	Rainbow trout	30
